# Association of body mass index with morbidity following elective ventral hernia repair^☆^

**DOI:** 10.1016/j.sopen.2023.06.005

**Published:** 2023-06-17

**Authors:** Russyan Mark Mabeza, Nam Yong Cho, Amulya Vadlakonda, Sara Sakowitz, Shayan Ebrahimian, Ashkan Moazzez, Peyman Benharash

**Affiliations:** a Cardiovascular Outcomes Research Laboratories (CORELAB), David Geffen School of Medicine, University of California, Los Angeles, CA, USA; b Depatment of Surgery, Harbor-UCLA Medical Center, Torrance, CA, USA

**Keywords:** Ventral hernia repair, Elective surgery, Body mass index, Outcomes

## Abstract

**Background:**

Prior work has linked body mass index (BMI) with postoperative outcomes of ventral hernia repair (VHR), though recent data characterizing this association are limited. This study used a contemporary national cohort to investigate the association between BMI and VHR outcomes.

**Methods:**

Adults ≥ 18 years undergoing isolated, elective, primary VHR were identified using the 2016–2020 American College of Surgeons National Surgical Quality Improvement Program database. Patients were stratified by BMI. Restricted cubic splines were utilized to ascertain the BMI threshold for significantly increased morbidity. Multivariable models were developed to evaluate the association of BMI with outcomes of interest.

**Results:**

Of ~89,924 patients, 0.5 % were considered *Underweight*, 12.9 % *Normal Weight*, 29.5 % *Overweight*, 29.1 % *Class I*, 16.6 % *Class II*, 9.7 % *Class III*, and 1.7 % *Superobese*. After risk adjustment, class I (Adjusted Odds Ratio [AOR] 1.22, 95 % Confidence Interval [95%CI]: 1.06–1.41), class II (AOR 1.42, 95%CI: 1.21–1.66), class III obesity (AOR 1.76, 95%CI: 1.49–2.09) and superobesity (AOR 2.25, 95 % CI: 1.71–2.95) remained associated with increased odds of overall morbidity relative to normal BMI following open, but not laparoscopic, VHR. A BMI of 32 was identified as the threshold for the most significant increase in predicted rate of morbidity. Increasing BMI was linked to a stepwise rise in operative time and postoperative length of stay.

**Conclusion:**

BMI ≥ 32 is associated with greater morbidity following open, but not laparoscopic VHR. The relevance of BMI may be more pronounced in open VHR and must be considered for stratifying risk, improving outcomes, and optimizing care.

**Key message:**

Body mass index (BMI) continues to be a relevant factor in morbidity and resource use for elective open ventral hernia repair (VHR). A BMI of 32 serves as the threshold for significant increase in overall complications following open VHR, though this association is not observed in operations performed laparoscopically.

## Introduction

Ventral hernia repair (VHR) is one of the most common general surgery procedures in the United States, with nearly 350,000 cases performed each year [1]. Prior work has estimated that a 1 % reduction in VHR stemming from decreased recurrence and postoperative complications, would yield ~$32 million in annual cost savings [1]. Nonetheless, the management of ventral hernias varies across institutions [2], posing significant challenges in minimizing mortality and morbidity following repair.

Several studies note obesity as a major risk factor for poor outcomes following VHR, including hernia recurrence, prolonged length of stay, and increased healthcare costs [[3], [4], [5]]. However, consensus regarding a suitable body mass index (BMI) threshold for not offering VHR is lacking [2]. Generally, a BMI δ30 has been considered safe for VHR, while elective repair is contraindicated in patients with BMI >50 [2]. Some institutional algorithms [6,7] recommend preoperative weight loss or bariatric surgery for obese candidates, but no weight loss targets have been established to date [8].

Given the significant number of VHR cases and associated complications, identification of potential risk factors is necessary to aid in surgical optimization and cost reduction. Furthermore, evolution in surgical technique, use of prosthetic materials, and advancements in perioperative management warrant a reexamination of a BMI threshold for improved patient selection and risk stratification in VHR. Using a contemporary national cohort, the present study characterized the association of BMI with VHR outcomes. We hypothesized that increasing BMI would be linked to increased morbidity, operative time, length of stay, and readmissions.

## Methods

**Fig. 1:**
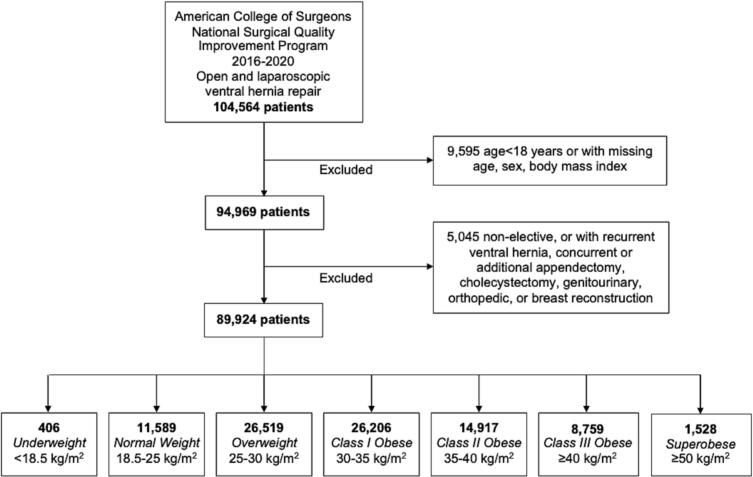
Patient selection criteria. Of the 94,969 ventral hernia repair patients identified, 89,924 (94.7 %) met inclusion criteria.

This retrospective study utilized the American College of Surgeons (ACS) National Surgical Quality Improvement Program (NSQIP) 2016–2020 participant use data files. Over 600 hospitals contribute to the NSQIP database to evaluate perioperative outcomes including 30-day morbidity and mortality. We identified all adults (≥18 years) undergoing elective open or laparoscopic repair of ventral hernia using relevant *Current Procedural Terminology* (CPT) primary procedure codes (Supplemental Table S1). Patients with missing data on age, sex, or BMI, hernia strangulation or incarceration, as well as those undergoing repair for recurrent ventral hernia or other concurrent procedures were excluded (Fig. 1).

Patients were stratified based on BMI class: <18.5 kg/m^2^ (*Underweight*), 18.5–24.9 kg/m^2^ (*Normal Weight*), 25–29.9 kg/m^2^ (*Overweight*), 30–34.9 kg/m^2^ (*Class I Obese*), 35–39.9 kg/m^2^ (*Class II Obese*), 40–49.9 kg/m^2^ (*Class III Obese*), and ≥50 kg/m^2^ (*Superobese*). Other patient characteristics included age, sex, race, functional status, comorbidities, and American Society of Anesthesiologists (ASA) class. Perioperative characteristics included operative approach, mesh placement, component separation, and preoperative serum laboratory values (Table 1). Patient and perioperative characteristics were defined according to the NSQIP data dictionary.

**Table 1:** Baseline patient and perioperative characteristics stratified by BMI class for patients undergoing ventral hernia repair.

Variable	Underweight	Normal weight	Overweight	Class I obese	Class II obese	Class III obese	Superobese	*P*-value
Age, years (median, IQR)	43 (33–53)	44 (33–54)	44 (34–53)	41 (32–50)	39 (31–48)	37 (29–45)	33 (25–42)	<0.001
Female (%)	76.6	59.8	42.6	45.5	53.6	63.5	70.4	<0.001
Race (%)								<0.001
White	77.3	77.2	76.0	74.7	74.1	72.0	71.6	
Black	7.4	7.7	8.4	9.8	11.9	14.4	16.7	
Asian/PI	3.2	2.4	1.6	1.1	0.9	0.7	0.4	
Other/unknown^a^	12.1	12.7	14.1	14.4	13.1	12.9	11.3	
Functional status (%)								<0.001
Independent	97.5	98.9	99.4	99.5	99.4	99.2	98.5	
Partially dependent	2.5	1.1	0.6	0.5	0.6	0.8	1.5	
Comorbidities (%)								
Ascites	0.3	0.7	0.3	0.2	0.1	0.1	0.1	<0.001
CHF	1.0	0.7	0.5	0.4	0.4	0.7	1.4	<0.001
COPD	12.3	6.0	4.3	4.3	4.2	4.5	5.1	<0.001
Diabetes mellitus	5.7	8.0	13.0	17.2	22.7	25.7	26.4	<0.001
Dialysis use	2.7	1.8	1.1	0.9	0.9	0.6	0.5	<0.001
Hypertension	32.0	37.0	46.1	51.0	55.5	56.8	57.1	<0.001
Smoking history	31.8	20.1	16.2	15.2	15.2	15.1	14.1	<0.001
ASA class (%)								<0.001
1	7.4	70.2	7.0	3.9	1.7	0.7	0.6	
2	40.6	49.0	52.4	53.4	45.8	26.1	14.8	
3	46.3	37.5	38.3	40.6	50.5	70.3	76.3	
4+	5.7	3.3	2.3	2.0	2.0	3.0	8.3	
Operative characteristics								
Open approach (%)	71.2	60.4	53.4	49.1	47.5	47.0	47.7	<0.001
Inpatient (%)	31.0	27.7	27.7	28.0	29.6	31.2	35.4	<0.001
Mesh placement	31.5	31.3	31.0	29.6	29.1	28.8	27.1	<0.001
Component separation	6.4	4.6	4.8	4.8	4.7	3.9	3.4	0.001
Preoperative serum values (median, IQR)								
Albumin, g/dL	3.9 ± 0.6	4.0 ± 0.5	4.1 ± 0.4	4.1 ± 0.4	4.1 ± 0.4	4.0 ± 0.4	3.9 ± 0.4	<0.001
HCT, %	39.2 ± 4.7	40.2 ± 4.1	41.2 ± 4.1	41.6 ± 4.0	41.5 ± 4.0	41.2 ± 3.9	40.7 ± 3.9	<0.001
Creatinine, mg/dL	1.0 ± 1.0	1.0 ± 1.0	1.1 ± 0.9	1.0 ± 0.8	1.0 ± 0.7	0.9 ± 0.6	0.9 ± 0.5	<0.001
BUN, mg/dL	18.1 ± 13.2	17.2 ± 9.8	17.3 ± 8.7	17.1 ± 8.9	16.7 ± 8.9	16.1 ± 8.0	15.4 ± 7.3	<0.001
Bilirubin, mg/dL	0.5 ± 0.2	0.5 ± 0.3	0.5 ± 0.3	0.5 ± 0.3	0.5 ± 0.3	0.5 ± 0.3	0.5 ± 0.4	<0.001

ASA, American Society of Anesthesiologists; BMI, body mass index; BUN, blood urea nitrogen; CHF, congestive heart failure; COPD, chronic obstructive pulmonary disease; IQR, interquartile range; HCT, hematocrit; PI, Pacific Islander; SD, standard deviation.

a Unknown indicates no recorded data for race.

The primary outcome of interest was overall morbidity while secondary endpoints included serious morbidity, operative time, postoperative length of stay (LOS), and 30-day readmission. Serious morbidity was defined as having a documented instance of the following: death, wound dehiscence, stroke, cardiac arrest, myocardial infarction, bleeding requiring transfusion, pulmonary embolism, prolonged mechanical ventilation, acute renal failure, and sepsis or septic shock. Overall morbidity was defined as having a documented instance of serious morbidity or the following additional postoperative complications: superficial SSI, deep SSI, organ space SSI, pneumonia, reintubation, urinary tract infection, and deep vein thrombosis.

Categorical variables are reported as frequency (%) while continuous variables are reported as mean with standard deviation (SD) or median and interquartile range (IQR). The chi-square and Kruskal Wallis tests were used to compare patient demographics, comorbidities, and outcomes by BMI class. Multivariable logistic and Poisson regression models were developed to evaluate the association of BMI class with outcomes of interest. Variable selection was guided by the least absolute shrinkage and selection operator (LASSO). Briefly, LASSO is an automated algorithm that reduces model overfitting and improves out-of-sample reliability [9]. We selected models to minimize the mean squared error term and evaluated them using receiver operating characteristic curves as well as Akaike and Bayesian information criteria, where appropriate. Final models included adjustment for demographics (age, sex, functional status), comorbidities (ascites, congestive heart failure, chronic obstructive pulmonary disease [COPD], and smoking history), clinical characteristics (operative approach, mesh placement, component separation, and ASA class), and preoperative serum laboratory values (serum creatinine and hematocrit). The Stata *margins* command was used to generate risk-adjusted outcomes with application of restricted cubic splines to ensure optimal fit and characterize potential thresholds. A sensitivity analysis was performed examining outcomes specific to patients who underwent mesh placement and component separation. A *P*-value <0.05 was considered statistically significant for all comparisons. All statistical analyses were performed using STATA 16.0 (StataCorp LP, College Station, TX).

## Results

Of the 104,564 ventral hernia patients identified, 89,924 (86.0 %) met inclusion criteria, of which 0.5 % were *Underweight*, 12.9 % *Normal Weight*, 29.5 % *Overweight*, 29.1 % *Class I Obese*, 16.6 % *Class II Obese*, 9.7 % *Class III Obese*, and 1.7 % *Superobese* (Fig. 1). The *Underweight* cohort was older (43 [33–53] vs. 33 [25–42] years, *P* < 0.001) but faced lower prevalence of diabetes (5.7 vs. 26.4 %, *P* < 0.001) and hypertension (32.0 vs. 57.1, *P* < 0.001) compared to *Superobese* (Table 1). However, underweight patients demonstrated higher prevalence of ascites, COPD, dialysis use, and smoking history (Table 1). A greater proportion of the *Underweight* cohort underwent open procedures (71.2 vs. 47.7 %, *P* < 0.001), mesh placement (31.5 vs. 27.1 %, P < 0.001), and component separation (6.4 vs. 3.4 %, P < 0.001) compared to the *Superobese* cohort (Table 1).

**Fig. 2:**
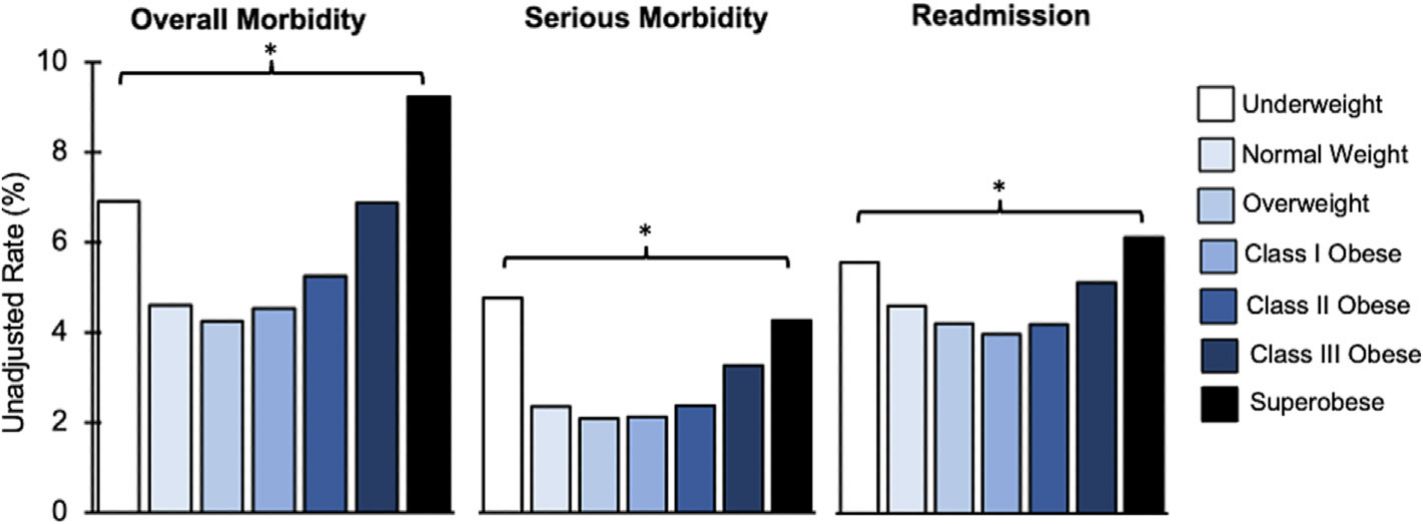
Unadjusted rates of overall morbidity, serious morbidity, and 30-day readmission by BMI class. *Indicates *P* < 0.05.

Unadjusted outcomes are shown in Table 2. Crude rates of overall morbidity demonstrated a U-shaped trend across BMI class, with the *Underweight* and *Superobese* cohorts having the highest proportion of adverse events (Fig. 2). A similar trend was observed for serious morbidity and 30-day readmission (Fig. 2). A stepwise decrease in rates of mortality was noted across BMI class, from 1.2 % among underweight patients to 0.1 % among those with superobesity (*P* < 0.001, Table 2). In addition, operative time increased in a stepwise manner from the *Underweight* to the *Superobese* cohort (60 [37–100] vs. 85 [55–132] minutes, P < 0.001, Table 2). Patients with superobesity experienced the longest postoperative LOS compared to the other cohorts (1 [0–2] days, Table 2).

**Table 2:** Unadjusted postoperative outcomes following elective ventral hernia repair by BMI class.

Variable	Underweight	Normal weight	Overweight	Class I obese	Class II obese	Class III obese	Superobese	*P*-value
Serious complications								
Mortality, %	1.2	0.4	0.2	0.2	0.2	0.2	0.1	<0.001
Wound dehiscence, %	1.0	0.2	0.2	0.3	0.3	0.5	0.6	<0.001
Stroke, %	0	0.08	0.05	0.05	0.05	0.03	0	0.81
Cardiac arrest, %	0	0.1	0.1	0.1	0.1	0.1	0.2	0.53
Myocardial infarction, %	0.5	0.1	0.2	0.2	0.1	0.2	0.1	0.37
Bleeding, %	1.7	0.7	0.6	0.4	0.5	0.5	0.8	<0.001
Pulmonary embolism, %	0.5	0.2	0.2	0.3	0.3	0.4	0.5	0.01
Prolonged ventilation, %	0.5	0.2	0.2	0.2	0.3	0.3	0.8	<0.001
Acute renal failure, %	0	0.1	0.2	0.2	0.3	0.4	0.9	<0.001
Sepsis or septic shock, %	1.0	0.7	0.5	0.5	0.5	0.9	1.3	<0.001
Other complications								
Superficial SSI, %	1.2	1.0	1.1	1.4	1.7	2.5	4.0	<0.001
Deep SSI, %	0.5	0.3	0.3	0.4	0.5	0.8	0.9	<0.001
Organ space SSI, %	0.3	0.5	0.5	0.6	0.5	0.8	1.3	<0.001
Pneumonia, %	1.0	0.7	0.6	0.6	0.5	0.5	1.1	<0.001
Reintubation, %	0.7	0.4	0.3	0.3	0.3	0.4	0.6	<0.001
Urinary tract infection, %	0.5	0.8	0.6	0.5	0.7	0.7	1.1	0.004
Deep vein thrombosis, %	0.3	0.2	0.2	0.3	0.3	0.3	0.3	0.34
Operative time, minutes	60 (37–100)	66 (39–107)	73 (45–117)	77 (48–122)	80 (50–127)	81 (53–127)	85 (55–132)	<0.001
Postoperative LOS, days	0 (0–2)	0 (0–1)	0 (0–1)	0 (0–1)	0 (0–2)	1 (0–2)	1 (0–2)	<0.001
30-day readmission, %	5.4	4.5	4.1	3.9	4.1	5.0	6.0	<0.001

Continuous variables reported as median with interquartile range. LOS, length of stay; SSI, surgical site infection.

**Fig. 3:**
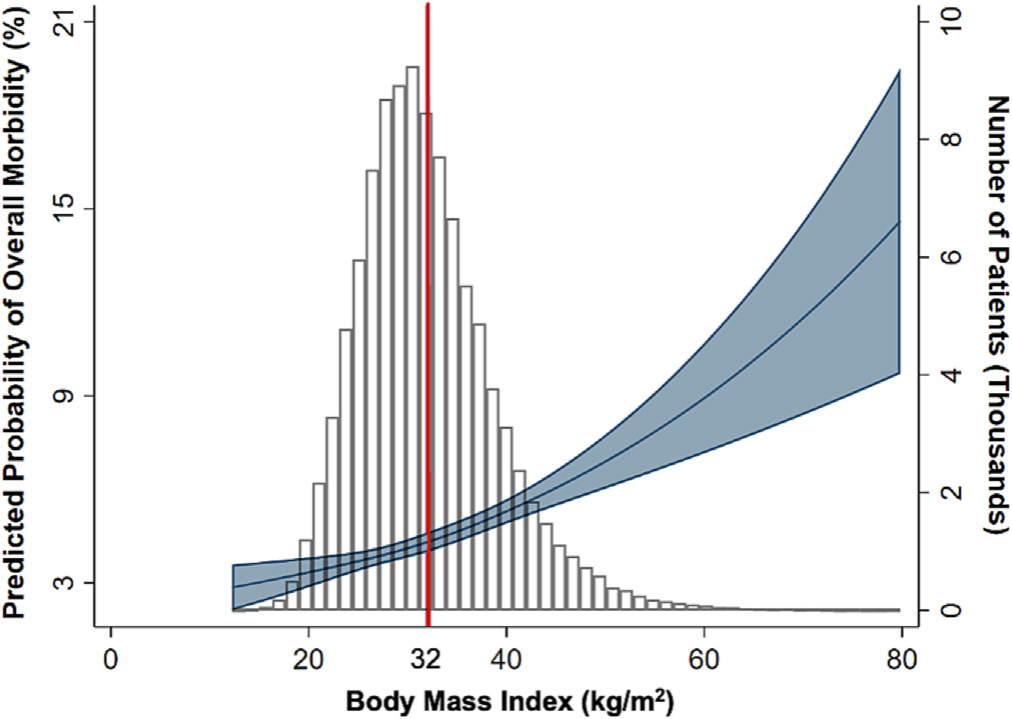
Spline analysis of risk-adjusted overall morbidity of open ventral hernia repair and number of patients by body mass index. A BMI of 32 displays a distinct maximum in change of slope for overall morbidity following ventral hernia repair.

**Fig. 4:**
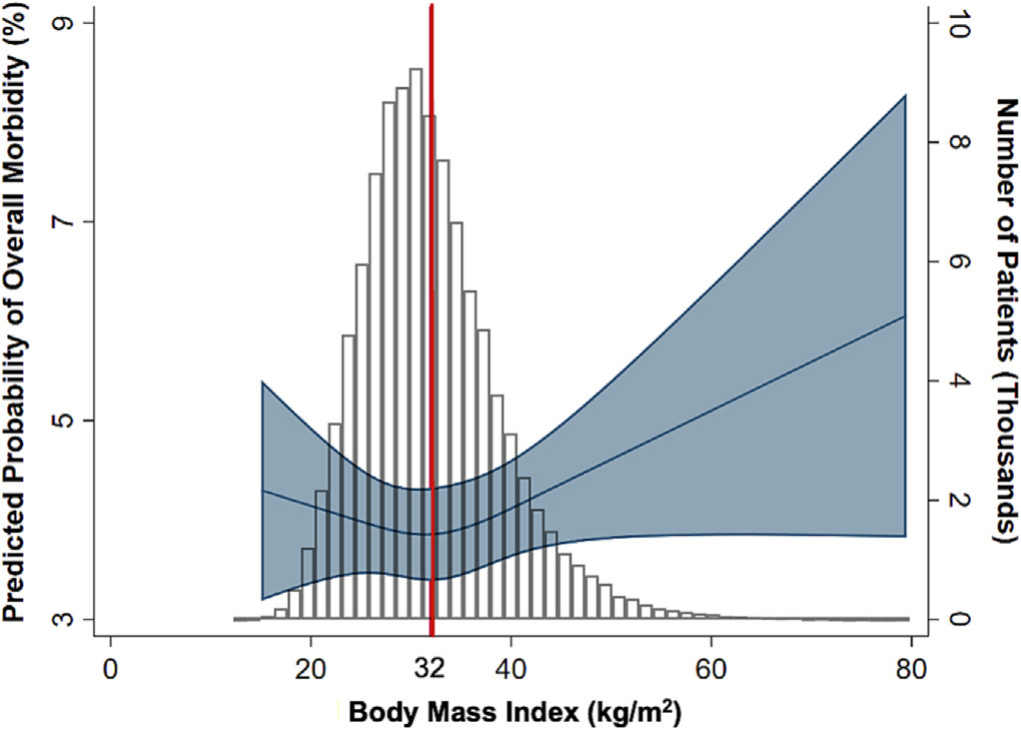
Spline analysis of risk-adjusted overall morbidity of laparoscopic ventral hernia repair and number of patients by body mass index. The predicted morbidity is higher among the extremes of BMI though these differences are not statistically significant.

After adjustment for patient and clinical characteristics, class I (Adjusted Odds Ratio [AOR] 1.22, 95 % Confidence Interval [CI]: 1.06–1.41), class II (AOR 1.42, 95 % CI: 1.21–1.66), class III (AOR 1.76, 95 % CI: 1.49–2.09), and superobesity (AOR 2.25, 95 % CI: 1.71–2.95) remained associated with increased odds of overall morbidity relative to normal weight following open VHR (Table 3). There was no significant difference in overall morbidity based on BMI among those that underwent the laparoscopic approach (Table 3). The plot of risk-adjusted rate of overall morbidity versus BMI demonstrated a distinct maximum in change of slope at approximately 32 kg/m^2^ for open VHR (Fig. 3). In contrast, predicted overall morbidity were higher among the extremes of BMI for laparoscopic cases, though these differences were not statistically significant (Fig. 4). Other factors significantly associated with overall morbidity following both operative approaches were age, dependent functional status, ASA class, component separation, and inpatient setting (Table 3).

**Table 3:** Adjusted multivariable regression of BMI class on overall morbidity following elective ventral hernia repair.

	*Open*		*Laparoscopic*	
	AOR	95 % CI	AOR	95 % CI
BMI class				
Underweight	1.43	0.89–2.27	0.92	0.33–2.60
Normal weight	Ref		Ref	
Overweight	1.06	0.91–1.22	0.94	0.75–1.16
Class I obese	1.22	1.06–1.41	0.87	0.70–1.08
Class II obese	1.42	1.21–1.66	1.02	0.81–1.28
Class III obese	1.76	1.49–2.09	1.01	0.78–1.31
Superobese	2.25	1.71–2.95	1.30	0.88–1.92
Age, per year	1.00	1.00–1.01	1.01	1.00–1.01
Female	1.00	0.92–1.10	1.18	1.03–1.35
Dependent functional status	1.85	1.39–2.47	1.57	0.99–2.47
Ascites	1.94	1.28–2.94	2.31	1.13–4.72
CHF	1.22	0.86–1.75	1.57	0.93–2.66
COPD	1.56	1.35–1.81	1.44	1.14–1.81
Smoking history	1.27	1.14–1.42	1.30	1.11–1.54
ASA class				
1	Ref		Ref	
2	1.38	0.93–2.05	2.09	1.14–3.84
3	1.97	1.32–2.93	3.31	1.80–6.10
4+	2.82	1.83–4.34	5.17	2.66–10.06
Mesh placement	0.96	0.88–1.05	1.34	0.93–1.94
Component separation	1.51	1.34–1.70	2.05	1.43–2.95
Inpatient	3.93	3.57–4.32	3.29	2.90–3.72
Preoperative laboratory values				
Creatinine, mg/dL	1.00	0.95–1.04	1.05	0.99–1.11
Hematocrit, %	0.97	0.96–0.98	0.98	0.97–1.00

ASA, American Society of Anesthesiologists classification; CI, confidence interval; COPD; chronic obstructive pulmonary disease; AOR: adjusted odds ratio.

Table 4 demonstrates additional adjusted outcomes following VHR stratified by open and laparoscopic. Underweight status (AOR 1.85, 95 % CI: 1.06–3.24), class II (AOR 1.30, 95 % CI: 1.05–1.62), class III (AOR 1.84, 95 % CI: 1.46–2.32), and superobesity (AOR 2.21, 95 % CI: 1.53–3.19) were associated with increased odds of serious morbidity relative to normal weight for open VHR. Such a difference in serious morbidity was not observed in laparoscopic cases (Table 4). For both approaches, increasing BMI was linked to a stepwise rise in operative time and postoperative LOS. Class III and superobesity were linked to significantly higher odds of readmission in open VHR but no significant difference was identified in patients undergoing vHR laparoscopically (Table 4).

**Table 4:** Adjusted postoperative outcomes following elective ventral hernia repair stratified by BMI class with Normal Weight as reference. Covariates include age, sex, functional status, ascites, congestive heart failure, chronic obstructive pulmonary disease, history of smoking, American Association of Anesthesiologists class, mesh placement, component separation, inpatient setting, preoperative creatinine, and preoperative hematocrit.

	Serious morbidity		Operative time		LOS		Readmission	
	AOR	95 % CI	β	95 % CI	β	95 % CI	AOR	95 % CI
Open								
Underweight	1.85	1.06–3.24	−5.8	−14.1–2.5	0.4	0.0–0.7	1.20	0.70–2.04
Overweight	1.00	0.82–1.21	5.7	3.6–7.8	0.1	−0.1 to 0.2	1.04	0.90–1.22
Class I obese	1.11	0.91–1.35	11.5	9.4–13.6	0.1	0.1–0.2	0.93	0.79–1.09
Class II obese	1.30	1.05–1.62	14.8	12.3–17.2	0.2	0.1–0.3	1.14	0.96–1.36
Class III obese	1.84	1.46–2.32	12.8	10.0–15.7	0.2	0.1–0.3	1.36	1.13–1.65
Superobese	2.21	1.53–3.19	19.5	14.1–24.9	0.7	0.4–0.9	1.58	1.15–2.17

Laparoscopic								
Underweight	0.97	0.23–4.11	−4.0	16.5–8.5	0.1	−0.3–0.5	0.90	0.36–2.26
Overweight	0.96	0.71–1.30	3.5	1.1–5.9	0.0	−0.1–0.1	0.81	0.67–0.97
Class I obese	0.96	0.71–1.30	5.3	2.9–7.6	0.1	−0.0–0.1	0.78	0.65–0.95
Class II obese	0.97	0.70–1.36	5.7	3.1–8.3	0.1	0.0–0.2	0.69	0.56–0.85
Class III obese	1.02	0.71–1.48	5.5	2.6–8.4	0.1	0.0–0.2	0.73	0.58–0.94
Superobese	1.40	0.80–2.44	4.5	−0.5 to 9.6	0.1	−0.1–0.2	0.69	0.45–1.06

Results of our sensitivity analysis are detailed in Supplemental Table S2. Among patients that underwent mesh placement, increasing BMI was associated with greater odds of overall morbidity after open but not laparoscopic VHR. Such findings are consistent with the overall study population. For those who underwent component separation in the setting of open VHR, the adjusted odds of overall morbidity were significantly higher in patients classified as *Class III* or *Superobese*. There was no significant association between BMI and overall morbidity among those that underwent component separation in the setting of laparoscopic VHR.

## Discussion

The prevalence of obesity has increased dramatically over the last three decades, now accounting for 42.4 % of adults in the United States [10,11]. With obesity as a known risk factor for hernia formation, elucidating the association between BMI and VHR outcomes is relevant to optimizing surgical treatment quality and efficiency [12,13]. In the present national study, we delineate a stepwise BMI-related rise in overall morbidity after open, but not laparoscopic, VHR. Furthermore, we identified a BMI threshold of 32 kg/m^2^ to confer the most significant increase in perioperative complications in those undergoing open VHR. Such a threshold was not identified in patients undergoing elective VHR laparoscopically. Increasing BMI was linked to a stepwise rise in operative time and postoperative length of stay for both operative approaches. Several of these findings warrant further discussion.

Obesity has been linked to greater morbidity in patients undergoing general emergency surgery, neurosurgical interventions, and lung resection procedures [[14], [15], [16]]. Furthermore, elevated BMI has been shown to be an independent predictor of wide-ranging complications such as acute coronary events and surgical site infection [7,17]. Among those undergoing VHR, Novitsky et al. showed the association between obesity and increased odds of delayed wound healing and severe pulmonary insufficiency [4]. In the present study, we found class I obesity and higher to be independently linked to higher rates of overall morbidity in open VHR. Notably, superobese individuals faced more than double the odds of experiencing a complication following this procedure. Only class II, III, and superobesity demonstrated a significant increase in odds of serious adverse events, suggesting a severity-dependent effect on complications following open VHR. The excess abdominal adipose tissue can impede proper tissue oxygenation and compromise respiratory function [18]. Noting a BMI ≥ 35 kg/m^2^ may lead to death, shock, or other fatal perioperative complications, severely obese patients may benefit from a weight loss intervention prior to VHR. Specifically, staged VHR followed by bariatric operations may offer an effective alternative to improve clinical outcomes in this population [19,20].

Variability exists regarding reported BMI thresholds for safety in elective VHR. In the analysis of an institutional cohort of patients undergoing ventral or incisional hernia repairs, Liu et al. found a BMI > 35.3 kg/m^2^ to be associated with increased hernia recurrence [21]. Another study of patients undergoing elective open ventral hernia repair in the NSQIP database found BMI > 24.2 kg/m^2^ to be the cutoff for surgical site infection vulnerability [22]. In the present study, we identified a BMI of 32 kg/m^2^ as the threshold for significant increase in perioperative morbidity in patients undergoing open VHR. Our findings add to existing literature aimed at refining evidence-based strategies for risk stratification in this growing population. Such insight may also aid in developing individualized approaches to minimize complications of VHR in obese patients.

Furthermore, operative approach may play a particularly important role in diminishing morbidity among those with high BMI. While BMI was linked to higher rates of complications among those with open VHR, we noted no such association among those that underwent the operation laparoscopically. This was similar to the findings by Henriksen et al., which demonstrated a BMI > 40 kg/m^2^ to be associated with a 6-fold increased risk for 90-day readmission in open VHR but not with minimally invasive surgery [23]. With the laparoscopic approach, cutting larger portions of tissue is generally avoided, which may lead to quicker postoperative recovery and fewer complications. It is also possible that patients undergoing laparoscopic VHR have less complicated hernias. However, due to often misdiagnosed defects and a higher likelihood of recurrence, the laparoscopic approach in obese patients may present with requirements of challenging technique and larger mesh implantation [24]. Nonetheless, in patients without previous hernia repair or a lateral defect, data regarding laparoscopic VHR appears to have advantages over the open approach in the obesity population.

In the current era of value-based care, there is a growing emphasis on delivering optimal care while minimizing healthcare costs. We found that advancing BMI class was associated with a stepwise increase in operative time and postoperative LOS. Severe obesity has been shown to prolong operative time and thus increase the risks of perioperative complications in gastroenterological operations [[25], [26], [27]]. Due to reduced access to the operative field, the greater force of retraction required, and more surfaces from which the patient may bleed, obese anatomy is more technically demanding compared to patients with normal BMI. These factors contribute to increased operative and recovery periods. As LOS is the major determinant of healthcare costs, increased duration of stay following ventral hernia repair highlights the financial burden of obesity [28]. In any case, the higher risk for morbidity and healthcare expenditure associated with increasing BMI must be weighed carefully against decreased quality of life and a potentially higher incidence of emergent surgery for incarceration or strangulation. Despite a significant increase in overall morbidity starting at BMI 32, the overall values are relatively low and may be within the acceptable range of postoperative complication risks. These findings may aid surgeons and patients in considering surgical risk while improving efficiency, optimizing resource use, and maximizing quality of care.

This study has several limitations. Since the NSQIP database is designed for quality improvement rather than survey purposes, our findings may not be nationally representative. In addition, granular hernia characteristics, including size, location, or type, could not be ascertained, and thus may have introduced sampling bias. Furthermore, information regarding mesh type, and failed non-operative management attempts could not be ascertained in the database. Lastly, long-term complications, such as recurrence rate, could not be evaluated as the NSQIP database provides data on 30-day postoperative outcomes and thus could not be defined as morbidity. Nonetheless, we utilized statistically validated methodologies to reduce bias and assess the association between body mass index classification and ventral hernia repair outcomes of interest.

In the present study, we identified BMI ≥ 32 kg/m^2^ to be associated with greater morbidity following elective VHR. Class II and III obesity was linked to higher odds of overall complications following open, but not laparoscopic, VHR. Patients at the extremes of the BMI spectrum (underweight status, class II, and class III obesity) faced significantly increased odds of severe adverse events. BMI continues to be a relevant predictor of operation duration and postoperative LOS. These findings highlight the continued need for individualized counseling and thorough surgical planning in elective VHR. While laparoscopic intervention or staged repair may benefit severely obese patients, a multidisciplinary approach with a nutritionist and occupational therapist for a weight loss program may further minimize morbidity in this common operation.

The following are the supplementary data related to this article.

Supplemental Table S1 *Current Procedural Terminology* (CPT) Codes for Identifying Study Population. Supplemental Table S1

Supplemental Table S2 Adjusted odds of overall morbidity following elective ventral hernia repair stratified by BMI class for patients that underwent mesh placement and component separation. Covariates include age, sex, functional status, ascites, congestive heart failure, chronic obstructive pulmonary disease, history of smoking, American Association of Anesthesiologists class, inpatient setting, preoperative creatinine, and preoperative hematocrit. *Indicates no patient included in the group. Supplemental Table S2

## Funding sources

None.

## Ethics approval

Not required. The study was deemed exempt from full review by the Institutional Review Board at the University of California, Los Angeles.

## CRediT authorship contribution statement

**Russyan Mark Mabeza:** Conceptualization, Methodology, Formal analysis, Writing – original draft. **Nam Yong Cho:** Formal analysis, Writing – review & editing. **Amulya Vadlakonda:** Formal analysis, Writing – review & editing. **Sara Sakowitz:** Writing – review & editing. **Shayan Ebrahimian:** Writing – review & editing. **Ashkan Moazzez:** Writing – review & editing. **Peyman Benharash:** Conceptualization, Writing – review & editing, Supervision.

## Declaration of competing interest

The authors have no related conflicts of interest to declare.
