# Supplementary material for: Association of body mass index with morbidity following elective ventral hernia repair
Source: Surg Open Sci. 2023 Jun 17;14:11–6. doi: 10.1016/j.sopen.2023.06.005 (PMC10319335; doi:10.1016/j.sopen.2023.06.005)
Supplement: Supplemental Table S1 — Current Procedural Terminology (CPT) Codes for Identifying Study Population. [file mmc1.docx]

**Supplemental Table S1.** *Current Procedural Terminology* (CPT) Codes for Identifying Study Population

| **Procedure** | **CPT Code** |
| --- | --- |
| Open ventral hernia repair | 49560, 49561, 49565, 49566 |
| Laparoscopic ventral hernia repair | 49652, 49653, 49654, 49655 |
