# Supplementary material for: Association of body mass index with morbidity following elective ventral hernia repair
Source: Surg Open Sci. 2023 Jun 17;14:11–6. doi: 10.1016/j.sopen.2023.06.005 (PMC10319335; doi:10.1016/j.sopen.2023.06.005)
Supplement: Supplemental Table S2 — Adjusted odds of overall morbidity following elective ventral hernia repair stratified by BMI class for patients that underwent mesh placement and component separation. Covariates include age, sex, functional status, ascites, congestive heart failure, chronic obstructive pulmonary disease, history of smoking, American Association of Anesthesiologists class, inpatient setting, preoperative creatinine, and preoperative hematocrit. *Indicates no patient included in the group. [file mmc2.docx]

**Supplemental Table S2.** Adjusted odds of overall morbidity following elective ventral hernia repair stratified by BMI class for patients that underwent mesh placement and component separation. Covariates include age, sex, functional status, ascites, congestive heart failure, chronic obstructive pulmonary disease, history of smoking, American Association of Anesthesiologists class, inpatient setting, preoperative creatinine, and preoperative hematocrit. *Indicates no patient included in the group.

|  | **Mesh Placement** | | | | **Component Separation** | | | |
| --- | --- | --- | --- | --- | --- | --- | --- | --- |
|  | **Open** | | **Laparoscopic** | | **Open** | | **Laparoscopic** | |
|  | AOR | 95% CI | AOR | 95% CI | AOR | 95% CI | AOR | 95% CI |
| Underweight | 1.56 | 0.80-3.10 | * | * | 1.89 | 0.65-5.49 | * | * |
| Normal Weight | Ref |  | Ref |  | Ref |  | Ref |  |
| Overweight | 1.13 | 0.93-1.38 | 0.77 | 0.21-2.81 | 0.97 | 0.68-1.40 | 0.55 | 0.17-1.83 |
| Class I Obese | 1.34 | 1.10-1.63 | 1.73 | 0.56-5.31 | 1.11 | 0.77-1.60 | 0.48 | 0.15-1.56 |
| Class II Obese | 1.41 | 1.14-1.75 | 0.82 | 0.21-3.18 | 1.43 | 0.96-2.12 | 0.72 | 0.20-2.61 |
| Class III Obese | 1.74 | 1.38-2.20 | 0.32 | 0.03-3.07 | 2.05 | 1.31-3.19 | 0.51 | 0.20-2.61 |
| Superobese | 2.71 | 1.87-3.91 | * | * | 3.72 | 1.80-7.71 | * | * |
